# Noninvasive Digital Detection of Fetal DNA in Plasma of 4-Week-Pregnant Women following *In Vitro* Fertilization and Embryo Transfer

**DOI:** 10.1371/journal.pone.0126501

**Published:** 2015-05-13

**Authors:** Bedri Karakas, Wafa Qubbaj, Saad Al-Hassan, Serdar Coskun

**Affiliations:** 1 Department of Molecular Oncology, King Faisal Specialist Hospital and Research Center, Riyadh, Saudi Arabia; 2 Department of Pathology and Laboratory Medicine, King Faisal Specialist Hospital and Research Center, Riyadh, Saudi Arabia; 3 Department of Obstetrics and Gynecology, King Faisal Specialist Hospital and Research Center, Riyadh, Saudi Arabia; 4 Alfaisal University, Riyadh, Saudi Arabia; Northeastern University, UNITED STATES

## Abstract

The discovery of cell-free fetal DNA (cfDNA) circulating in the maternal blood has provided new opportunities for noninvasive prenatal diagnosis (NIPD). However, the extremely low levels of cfDNA within a high background of the maternal DNA in maternal circulation necessitate highly sensitive molecular techniques for its reliable use in NIPD. In this proof of principle study, we evaluated the earliest possible detection of cfDNA in the maternal plasma by a bead-based emulsion PCR technology known as BEAMing (beads, emulsion, amplification, magnetics). Blood samples were collected from in vitro fertilization (IVF) patients at 2 to 6 weeks following embryo transfer (i.e., 4 to 8 week pregnancies) and plasma DNA was extracted. The genomic regions of both X and Y chromosome-specific sequences (AMELX and AMELY) were concurrently amplified in two sequential PCRs; first by conventional PCR then by BEAMing. The positive beads either for AMELX or AMELY gene sequences were counted by a flow cytometer. Our results showed that the pregnancies yielding boys had significantly higher plasma AMELY gene fractions (0.512 ± 0.221) than the ones yielding girls (0.028 ± 0.003) or non-pregnant women (0.020 ± 0.005, P= 0.0059). Here, we clearly demonstrated that the BEAMing technique is capable of reliably detecting cfDNA in the blood circulation of 4-week-pregnant women, which is only two weeks after the embryo transfer. BEAMing technique can also be used to early detect fetal DNA alterations in other pregnancy-associated disorders.

## Introduction

The rare inherited disorders are observed throughout the world, especially in the societies with high rates of consanguinity. As of May 2013, there were over 3,800 disorders with known phenotype and genetic mechanisms reported in the public database, Online Mendelian Inheritance in Men (OMIM: http://www.omim.org/statistics/entry). The management of these disorders in affected children incurs high financial cost in health care systems and emotional burden on families. The most viable solution in circumventing these problems is through preventive strategies such as pre-implantation genetic diagnosis (PGD) following in vitro fertilization (IVF) and prenatal diagnosis (PND) in spontaneous pregnancies [1]. The invasive PND techniques have been utilized in the form of chorionic villus sampling and amniocentesis since 1960s [2,3]. However, due to the possible harmful effects on fetal development, non-invasive approaches have been sought in detecting fetal cells [4] and/or fetal nucleic acids [5,6] in maternal blood circulation.

In recent years, blood circulating nucleic acids (CNA) have been utilized as biomarkers for noninvasive monitoring tumor response to therapy in oncology as well as detecting fetal genetic abnormalities in PND [7–11]. Although CNA was discovered more than 60 years ago, their true diagnostic value has been only recently realized [12]. With the advancement of molecular genetics technologies in 1970s, many attempts have been made to associate the total or altered blood CNA with the disease phenotype [13,14].

The diagnostic potentials of cell-free fetal DNA (cfDNA) in maternal blood circulation have been investigated extensively [15] with the increasingly sensitive molecular technologies such as real-time PCR [16], digital PCR [10] and next-generation sequencing [17]. Since its discovery in 1985, many variations of PCR have been developed of which the most sensitive is emulsion PCR technique [7,18]. In emulsion PCR, millions of independent PCR amplifications take place within 3 to 5 micron diameter in size oil droplets, each harboring a single DNA molecule.

The non-invasive prenatal diagnosis (NIPD) of fetal genetic abnormalities in the earliest possible stage is crucial for successful intervention and/or treatment during fetal development [1]. Highly sensitive digital technologies are capable of reliably identifying cfDNA within a high background of maternal DNA in the maternal plasma [10, 17,19]. However, the capabilities of these technologies need to be carefully evaluated in the clinical settings with appropriate controls.

In this proof of principle study, we evaluated a digital PCR technology called BEAMing (beads, emulsion, amplification, magnetics), for detecting male cfDNA from the maternal plasma by targeting Y chromosome-specific sequences of the amelogenin gene (AMELY).

## Materials and Methods

### Ethics Statement

This study was approved by the Research Ethics Committee (REC) of the King Faisal Specialist Hospital and Research Centre (KFSH&RC) and all clinical investigation have been conducted according to the principles expressed in the Declaration of Helsinki. Patients agreed to participate in the study were properly informed and their written consents were obtained.

### Patient Selection and Blood Sample Collection

Women who visited the KFSH&RC In Vitro Fertilization (IVF) Clinic seeking treatment were recruited. A total of 16 blood samples from pregnant women at 2 to 6 weeks following the embryo transfer (i.e., 4 to 8 weeks of pregnancy) were collected. Another set of blood samples (n = 10) from non-pregnant nulliparous women (n = 10) was also obtained using them as plasma negative controls for the study.

### Plasma DNA Extraction

Fresh blood samples were drawn into BD Vacutainer EDTA tubes (Cat# 366643), immediately spun at 3,000 rpm for 10 min in a refrigerated centrifuge; their plasma fractions were collected and stored at -80°C for further analysis. Plasma CNA was extracted using QIAamp Circulating Nucleic Acid Kit (Qiagen, Cat# 55114) by following manufacturer’s protocol. Briefly, 2 ml of plasma was mixed with 200 μl Proteinase K, 2.4 ml buffer ACL by pulse-vortexing for 30 seconds in a 50 ml conical tube. Then the mixture was incubated in a 60°C water bath for 30 minutes. Then, 3.6 ml ACB buffer was added to the sample lysate and mixed by pulse-vortexing for 30 seconds, incubated for 5 min on ice and applied to the QIAamp mini column. Following the multiple wash and centrifugation steps, columns were incubated for 10 min at 56°C, DNA was eluted with 100 μl of buffer AVE and stored at -20°C for further analyses.

### Preamplification and BEAMing

We performed two sequential PCR amplifications for the digital quantification of cfDNA in the maternal plasma; 1^st^ PCR (preamplification) and 2^nd^ PCR (BEAMing). First, we used primers that concurrently amplify genomic regions of both AMELX and AMELY genes in the preamplification step. Then, we used AMELY and AMELY gene specific primers each with a unique tail sequence in the BEAMing reaction. All the primer and probe sequences used in both amplification steps are listed in Table 1.

**Table 1:** All the primers and probes used in the study.

Name	Sequence*	Type
**Tag1** ^**a**^	TCCCGCGAAATTAATACGAC	5’ double-biotinylated forward primer used in BEAMing; forward primer adaptor sequence used in preamplification step
**Tag2** ^**b**^	GCTGGAGCTCTGCAGCTA	Adaptor sequence for the reverse primer used in preamplification step
AMELX/Y-F	TCCCGCGAAATTAATACGACGGGCTCTGTAAAGAATAGTG	AMELX/Y specific forward primer with Tag1 adaptor used in the preamplification rstep (AMELX^**c**^ and AMELY^**d**^)
AMELX/Y-R	GCTGGAGCTCTGCAGCTATGGGAAGCTGATGGTAGGA	The AMELX allele specific reverse primer used in the BEAMing reaction (amplicon size = 50 bp)
AMELX-R	GAATTGCCGTTACTCGCTTGAGAAACATCTGGGATA	The AMELX allele specific reverse primer used in the BEAMing reaction (amplicon size = 50 bp)
AMELY-R	TCCAGCCTAACAACGTCAACCACTTTATTTGGGATG	AMELY allele specific reverse primer used in the BEAMing reaction (amplicon size = 50 bp)
AMELX-FITC	GAATTGCCGTTACTCGC	5’ FITC-labeled probe specific to the AMELX tail sequence
AMELY-Cy5	TCCAGCCTAACAACGTC	5’ Cy5-labled probe specific to the AMELY tail sequence

^**a**^Bold nucleotides denote gene specific sequences.

^**b**^Diehl et al 2006.

^**c**^ChrX: 11296878–11296965

^**d**^ChrY: 6869861–68619954

The preamplification PCR was performed in 45 μl reaction mix that included a high fidelity DNA polymerase (Phusion polymerase, Cat. #M0530, NEB) and 10 or 30 ng cellular or plasma DNA, respectively. The preamplification reactions were performed in the following thermocycler settings: (98°C for 30 sec) x 1 cycle; (98°C for 10 sec, 60°C for 30 sec, 72°C for 30 sec) x 35 cycles; and (72°C for 5 min) x 1 cycle.

**Fig 1:**
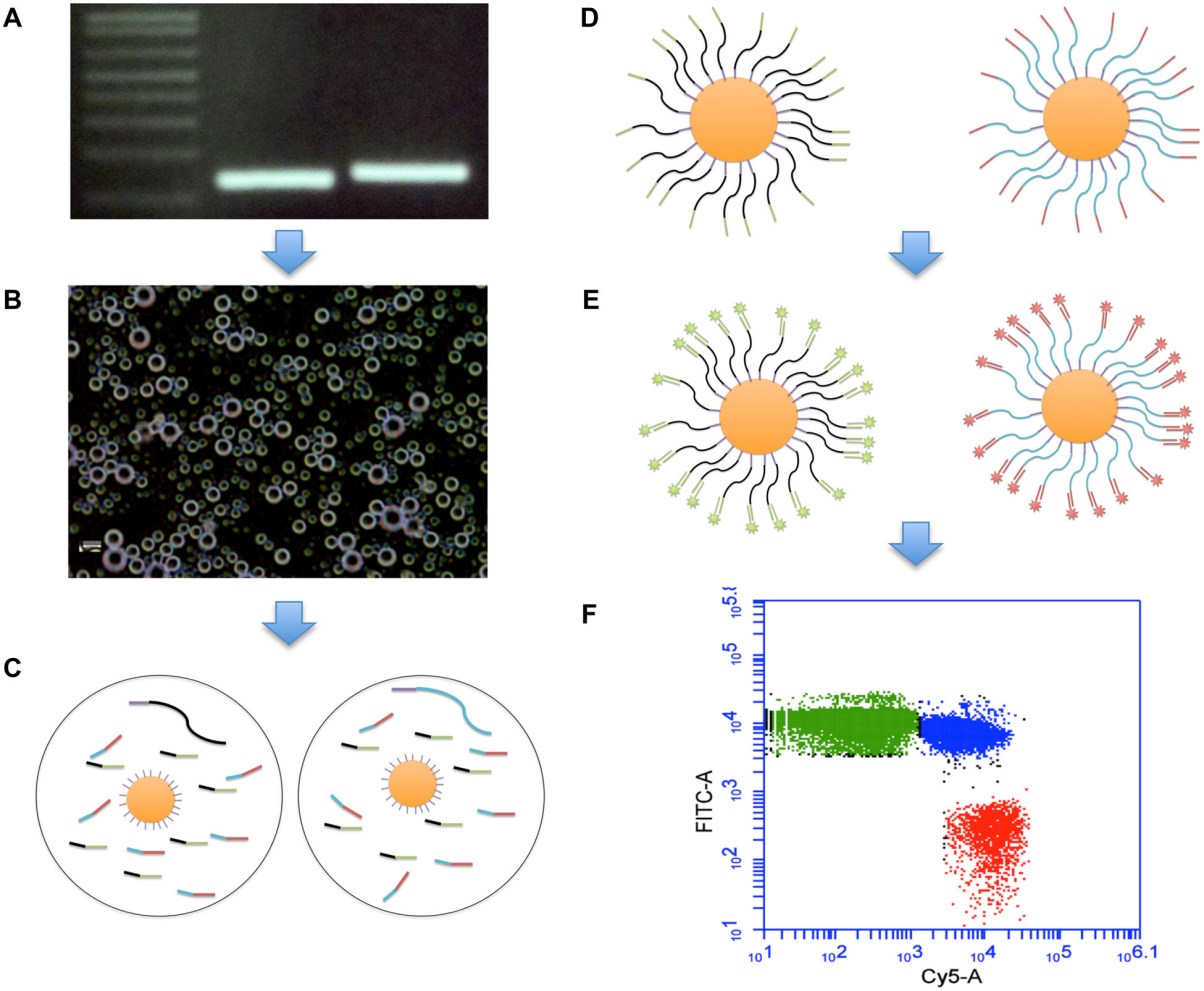
An overview of the BEAMing protocol. (**A**) AMELX and AMELY gene products from the preamplification step, (**B**) water-in-oil emulsion droplets that include PCR reaction mix with allele specific primers for AMELX and AMELY genes, a single DNA molecule from the preamplification step, and a single primer-coated magnetic bead, (**C**) oil droplets each harboring either the AMELX or AMELY gene fragment, (**D**) beads coated with either AMELX or AMELY-specific DNA following the BEAMing reaction, (**E**) beads hybridized either with the Cy5-AMELY or FITC-AMELX probe, (**F**) flow cytometry result of a test sample. Green beads represent AMELX, red beads AMELY, and blue beads represent both AMELX and AMELY-specific DNA sequences.

The BEAMing technique is based on clonal amplifications of single DNA molecules on millions of primer-coated magnetic beads within 3 to 5 micron diameter in size oil emulsions followed by flow cytometric analysis of the DNA coated beads (Table 1, Fig 1A–1F, Fig in S1 Fig). A typical BEAMing reaction mixture is prepared as follows; 16 μl 10X PCR buffer (670 mM Tris HCL (pH 8.8), 166 mM (NH_4_)_2_ SO_4_, 100 mM βMercaptoethanol, 67 mM MgCl_2_), 3.2 μl dNTP mix (10 mM), 3.2 μl forward primer (2.5 μM), 6.4 μl beads (Dynabeads MyOne Streptavidin C1, Cat # 650.01, Invitrogen) coated with Tag1 forward primer, 3.2 μl (100 μM) AMELX specific reverse primer, 3.2 μl (100 μM) AMELY specific reverse primer, 10 μl Taq polymerase (5 U/ μl) (Cat #18038, Invitrogen) and 15 pg of DNA from preamplification step. All BEAMing reactions were carried out under the following thermocycler settings: (94°C for 2 min) x 1 cycle; (68°C for 45 sec, 70°C for 60 sec) x 2 cycles; (65°C for 45 sec, 70°C for 60 sec) x 2 cycles; (61°C for 45 sec, 70°C for 60 sec) x 2 cycles; (58°C for 45 sec, 70°C for 60 sec) x 2 cycles; (55°C for 45 sec, 70°C for 60 sec) x 50 cycles.

Following the completion of BEAMing reaction, beads were processed as previously described [20]. Briefly, oil emulsions were broken open and DNA-coated beads were magnetically captured and hybridized with the AMELX and AMELY tail sequence-specific probes, each labeled with a unique fluorophore (Table 1, Fig in S1 Fig). The amplified and hybridized beads were analyzed using BD Accuri C6 Flow Cytometer (Cat #653118, BD Biosciences) by counting AMELX and AMELY specific beads. Up to 200,000 positive beads were counted in proportional with the number of AMELY positive fractions for each sample (Figs A–E in S2 Fig). For example, more beads were analyzed for the plasma samples that contained low AMELY whereas fewer beads were counted for the ones with high AMELY fractions. The relative AMELY fractions for all the samples were calculated as follows: ((number of AMELY-positive beads) / (number of AMELY-positive beads + AMELX-positive beads)) x 100. The statistical differences between the plasma AMELY fractions of the women who delivered boys and the AMELY fractions of the women who delivered girls or nulliparous women was calculated by one-way ANOVA with a *P*-value of <0.05 significance level. Tukey’s HSD test was used to determine the statistical differences between groups. An overview of the BEAMing protocol is depicted in Fig 1A–1F.

## Results

### BEAMing estimates known DNA amounts with high accuracy

**Fig 2:**
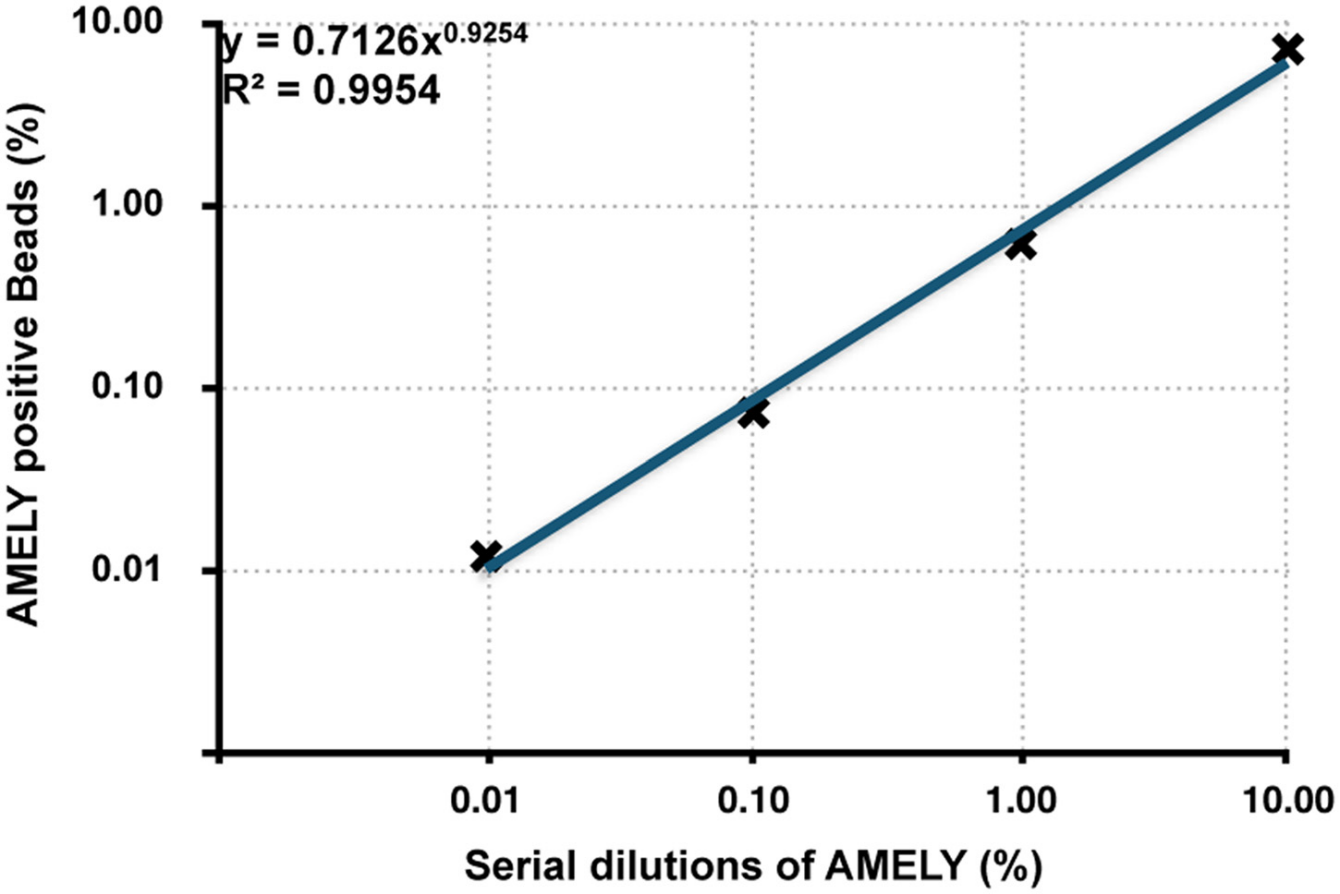
The sensitivity and accuracy of the BEAMing technique. Human genomic DNA from a male subject (AMELY) is serially diluted with the female DNA (AMELX) in concentrations of 10%, 1%, 0.1%, and 0.01% AMELY (r^2^>0.99).

First, we wanted to determine the sensitivity and accuracy of the BEAMing technique on spike-in control samples. To achieve this, we mixed known amounts of the AMELY (XY; male) with the AMELX (XX; female) and obtained a10-fold dilution series of the AMELY gene (10% to 0.01%). We then applied the BEAMing assay on these serially diluted samples containing a range of AMELY concentrations, and found a strong correlation between the counted AMELY-positive bead fractions and AMELY sample concentrations (r^2^ > 0.99) (Fig 2, Figs A–E in S2 Fig). With the BEAMing, we were able to detect the AMELY gene fragments as low as in 0.01% frequencies.

### Low Biological Background

**Fig 3:**
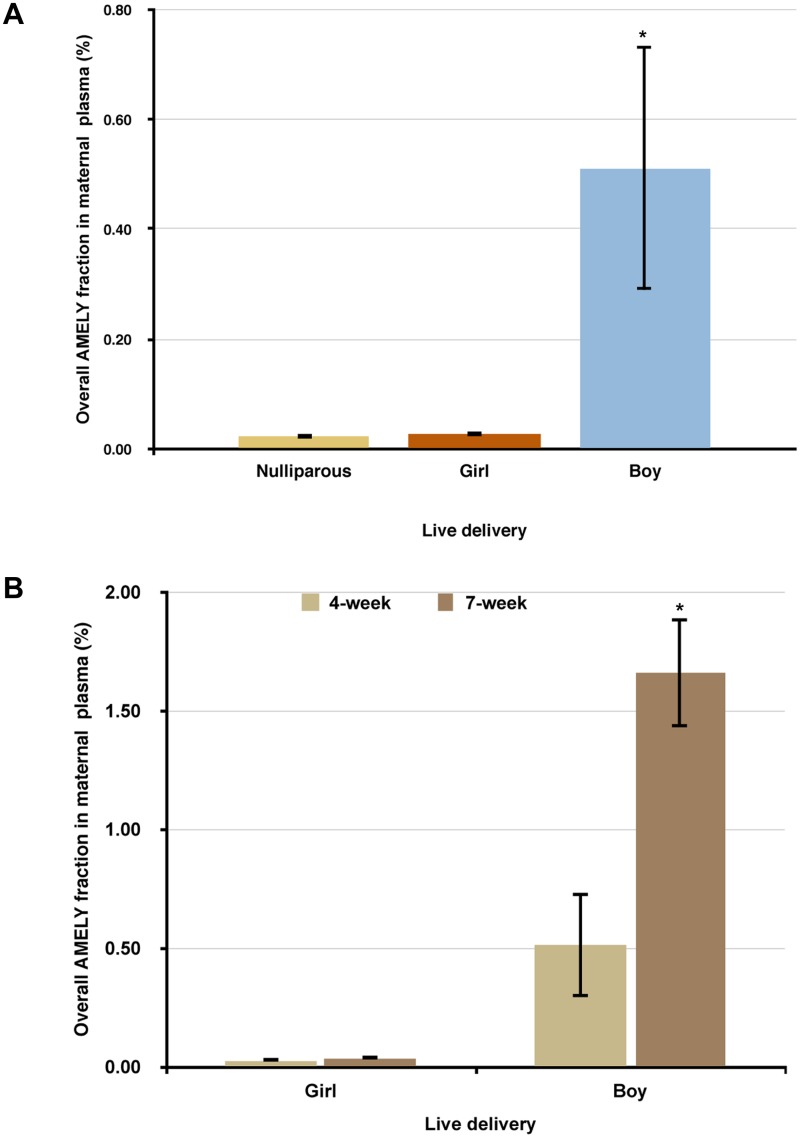
**(A)** The mean and standard error (SE) of the AMELY gene fractions from plasma samples of the nulliparous women (n = 10), the 4-week-pregnant mothers with girl deliveries (n = 4), and the 4-week-pregnant mothers with boy deliveries (n = 5). **(B)** The mean and standard error (SE) of the AMELY gene fractions of the plasma samples of 4 or 7 week-pregnant women with either girl or boy deliveries. *Statistically significant (P<0.05).

We next determined the plasma AMELY levels of non-pregnant nulliparous women (n = 10) using them as negative controls for any biological background. We found the mean and standard error of AMELY- specific bead fractions to be 0.020 ± 0.005, indicating a minimal background for the negative plasma control samples (Fig 3A).

### BEAMing reliably detected fetal DNA in plasma from 4-week pregnant women

**Fig 4:**
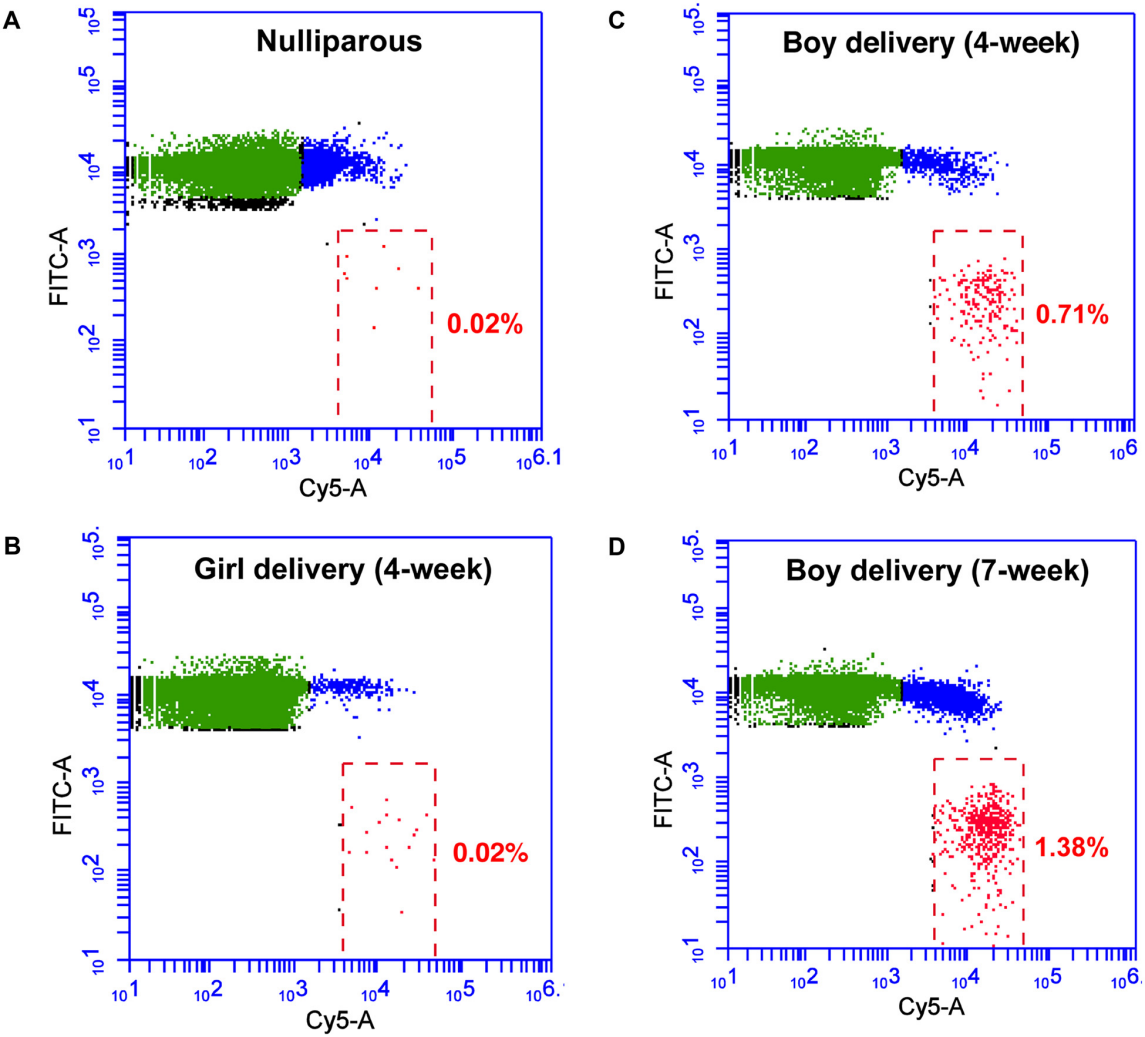
The representative flow cytometric profiles showing the plasma AMELY fractions (red beads) of (A) a nulliparous woman, (B) a 4-week-pregnant mother who delivered a girl, (C) a 4-week-pregnant mother who delivered a boy, (D) a 7-week-pregnant mother who delivered a boy. Sample names and percent AMELY positive fractions are shown on each plot.

Among the 9 pregnant women whose blood samples collected during the first trimester, 4 women delivered girls while 5 delivered boys. The plasma AMELY-specific bead fractions of the blood samples taken at the 4^th^ week of the pregnancy for the women either delivering girls or boys were 0.028 ± 0.003 or 0.512 ± 0.221, respectively. At the 4^th^ week of pregnancy, blood samples from the women who delivered boys had a significantly higher AMELY fraction than the ones taken from the women who delivered girls or nulliparous women (*P* = 0.0059, Fig 3A). While the percent AMELY fraction significantly increased for the pregnancies yielding boys as the pregnancy advanced from 4 weeks to 7, it remained identical for the pregnancies yielding girls (Fig 3B). Although our data showed an overall statistical significance between the boy and girl deliveries, one sample collected 15 days after the embryo transfer from a woman who delivered a boy had very low plasma AMELY, which was similar to the levels of those women with girl deliveries. A representative flow cytometry profile for each sample category showing the plasma AMELY fractions is included in Fig 4A–4D.

## Discussion

Our results indicate that it is possible to reliably detect and enumerate cfDNA from the maternal blood of 4-week-pregnant women by the BEAMing technique. To our knowledge, previous to this work, the earliest reliable cfDNA detection time point was 7 weeks in pregnancy [21].

Using the BEAMing assay on cfDNA from the maternal blood plasma, we have accurately identified the male gender in 4 out of 5 pregnancies (80%) on average 15.4 days following the embryo transfer. The low AMELY fraction in one sample taken 15 days after embryo transfer from a woman who delivered a boy could be due to the nature of the sample itself rather than the technical capabilities of the BEAMing assay. We believe that failure to accurately determine the gender in this sample is due to the kinetics of fetal DNA appearance in this patient.

In an earlier study, Guibert et al. (2003) detected cfDNA (SRY gene) in the maternal blood samples taken 18 days after the embryo transfer by a quantitative real-time PCR assay [19]. However, their overall efficiency was 33% in the first blood sampling stage with on average of 20.2 days. Moreover, the SRY gene was detectable only in one out of 10 samples within 18 days. The same authors have also reported to reach 80% detection levels of the cfDNA 27 days after the embryo transfer [19]. In our study, however, we have reached 80% detection on average 15.4 days after the embryo transfer, thus demonstrating higher sensitivity levels of the BEAMing technology compared to other conventional PCR techniques.

The high sensitivity of the BEAMing assay is based on the amplification of single DNA molecules within separate oil compartments in a massively parallel scale (Fig 1A–1F) [20]. Therefore, using BEAMing, one can detect extremely rare DNA molecules in a background of the more common ones due to the clonal amplification of each DNA molecule within separate oil compartments. This digital PCR technique can be easily performed by any trained technical staff in a molecular biology laboratory equipped with a homogenizer or tissue lyser, thermocycler, and a flow cytometer.

The BEAMing technique was originally developed to detect circulating tumor DNA in the plasma of colorectal cancer patients for a noninvasive and more sensitive monitoring of the treatment response to therapy [8, 22]. In this proof of concept work, we have addressed the feasibility of using this sensitive technology for earlier detection of the cfDNA in NIPD. However, a study with a larger sample size is needed before it can be routinely used in the clinic. If validated in the future clinical studies and approved, using BEAMing in the NIPD settings could ease the decision making-process of the pregnant women, whether to stop the pregnancies in the case of fetuses with genetic defects. Moreover, the psychological and emotional impact of the abortion on pregnant women will be less if performed soon after a positive pregnancy test. Similarly, health care professionals could plan for any available in utero treatment options.

## Conclusions

We demonstrated that the BEAMing technique is capable of reliably detecting cfDNA in the blood circulation of 4-week pregnant women (two weeks following embryo transfer). This highly sensitive digital assay can also be used in the noninvasive and early detection of the fetal DNA alterations of other pregnancy-associated disorders for possible interventions or terminating pregnancies.

## Supporting Information

S1 Fig A typical flow cytometric profile of the BEAMing protocol. Beads are separated by a flow cytometer as negative (unamplified), AMELX positive, AMELY positive and double positives (AMELX and AMELY). The beads, positive only for a single signal type (AMELX, green beads, or AMELY, red beads) are used for calculations; beads with both AMELY and AMELY DNA (double positives, blue beads) are excluded from the analyses.
(PDF)

S2 Fig The Flow cytometry results of the serially diluted AMELY samples. The relative AMELY positive fractions of serially diluted samples; (**A)** 0.0%, (**B)** 0.01%, (**C)** 0.1%, (**D**) 1%, and (**E)** 10%.
(PDF)

## References

[1] Bianchi DW. From prenatal genomic diagnosis to fetal personalized medicine: progress and challenges. Nat Med. 2011; 18: 1041–1051. 10.1038/nm.2829 PMC4433004 22772565

[2] Ferguson-Smith MA, Bianchi DW. Prenatal Diagnosis: past, present, and future. Prenat Diagn. 2010;30: 601–604. 10.1002/pd.2574 20572116

[3] Resta RG. The first prenatal diagnosis of a fetal abnormality. J Genet Counsel. 1997;6: 81–84. 10.1023/A:1025616019552 26141963

[4] Herzenberg LA, Bianchi DW, Schröder J, Cann HM, Iverson M. Fetal cells in the blood of pregnant women: detection and enrichment by fluorescence-activated cell sorting. Proc Natl Acad Sci USA. 1979;76: 1453–1455. 286330 10.1073/pnas.76.3.1453 PMC383270

[5] Lo YMD, Corbetta N, Chamberlain PF, Rai V, Sargent IL, Redman CWG, et al Presence of fetal DNA in maternal plasma and serum. The Lancet. 1997;350: 485–487. 9274585 10.1016/S0140-6736(97)02174-0

[6] Poon LLM, Leung TN, Lau TK, Lo YMD. Presence of fetal RNA in maternal plasma. Clin Chem. 2000;46: 1832–1834. 11067820

[7] Dressman D, Yan H, Traverso G, Kinzler KW, Vogelstein B. Transforming single DNA molecules into fluorescent magnetic particles for detection and enumeration of genetic variations. Proc Natl Acad Sci USA. 2003;100(15): 8817–8822. 12857956 10.1073/pnas.1133470100 PMC166396

[8] Diehl F, Schmidt K, Choti MA, Romans K, Goodman S, Li M, et al Circulating mutant DNA to assess tumor dynamics. Nat Med. 2007;14: 985–990. 10.1038/nm.1789 18670422 PMC2820391

[9] Lippman M, Osborne CK. Circulating Tumor DNA—Ready for Prime Time? N Eng J Med. 2013;368(13): 1249–1250. 10.1056/NEJMe1301249 23484798

[10] Lo YMD, Lun FMF, Chan KCA, Tsui NBY, Chong KC, Lau TK, et al Digital PCR for the molecular detection of fetal chromosomal aneuploidy. Proc Natl Acad Sci USA. 2007;104: 13116–13121. 17664418 10.1073/pnas.0705765104 PMC1934923

[11] Zimmermann BG, Grill S, Holzgreve W, Zhong XY, Jackson LG, Hahn S. Digital PCR: a powerful new tool for noninvasive prenatal diagnosis? Prenat Diagn. 2008;28: 1087–1093. 10.1002/pd.2150 19003785

[12] Mandel P, Metais P. Les acides nucleiques du plasma sanguine chez I’homme. Comptes Rendus de l’Académie des Sciences. 1948;142:241–243. 18875018

[13] Leon SA, Shapiro B, Sklaroff DM, Yaros MJ. Free DNA in the serum of cancer patients and the effect of therapy. Cancer Research. 1977;37: 646–650. 837366

[14] Castells A, Puig P, Móra J, Boadas J, Boix L, Urgell E, et al K-ras mutations in DNA extracted from the plasma of patients with pancreatic carcinoma: diagnostic utility and prognostic significance. Journal of Clinical Oncology. 1999;17: 578–584. 10080602 10.1200/JCO.1999.17.2.578

[15] Simpson SL. Cell-free fetal DNA and maternal serum analytes for monitoring embryonic and fetal status. Fertility and Sterility. 2013;99: 1124–1134. 10.1016/j.fertnstert.2013.02.012 23499003

[16] Lo Y, Leung TN, Tein MSC, Sargent IL, Zhang J, Lau TK, et al Quantitative abnormalities of fetal DNA in maternal serum in preeclampsia. Clinical Chemistry. 1999;45(2): 184–188. 9931039

[17] Forshew T, Murtaza M, Parkinson C, Gale D, Tsui DWY, Kaper F, et al Noninvasive identification and monitoring of cancer mutations by targeted deep sequencing of plasma DNA. Science Transl Med. 2012;4(136): 136ra68 10.1126/scitranslmed.3003726 22649089

[18] Tawfik DS & Griffiths AD. Man-made cell-like compartments for molecular evolution. Nature Biotechnology. 1998;16(7): 652–656. 9661199 10.1038/nbt0798-652

[19] Guibert J, Benachi A, Grebille AG, Ernault P, Zorn JR, Costa JM. Kinetics of SRY gene appearance in maternal serum: detection by real-time PCR in early pregnancy after assisted reproductive technique. Human Reproduction. 2003;18(8): 1733–1736. 12871892 10.1093/humrep/deg320

[20] Diehl F, Li M, He Y, Kinzler KW, Vogelstein B, Dressman D. BEAMing: single-molecule PCR on microparticles in water-in-oil emulsions. Nat Meth. 2006;3: 551–559. 10.1038/nmeth898 16791214

[21] Fernández-Martínez FJ, Galindo A, Garcia-Burguillo A, Vargas-Gallego C, Nongues N, Moreno-Garcia M, et al Noninvasive fetal sex determination in maternal plasma: a prospective feasibility study. Genet Med. 2012;14: 101–106. 10.1038/gim.2011.8 22237438

[22] Higgins MJ, Jelovac D, Barnathan E, Blair B, Slater S, Powers P, et al Detection of tumor PIK3CA status in Metastatic Breast Cancer using Peripheral Blood. Clin Cancer Res. 2012;18(2): 3462–3469. 22421194 10.1158/1078-0432.CCR-11-2696 PMC3533370

