# Supplementary figures and images for: Noninvasive Digital Detection of Fetal DNA in Plasma of 4-Week-Pregnant Women following In Vitro Fertilization and Embryo Transfer
Source: PLoS One. 2015 May 13;10(5):e0126501. doi: 10.1371/journal.pone.0126501 (PMC4430227; doi:10.1371/journal.pone.0126501)

Supporting Information File 1.

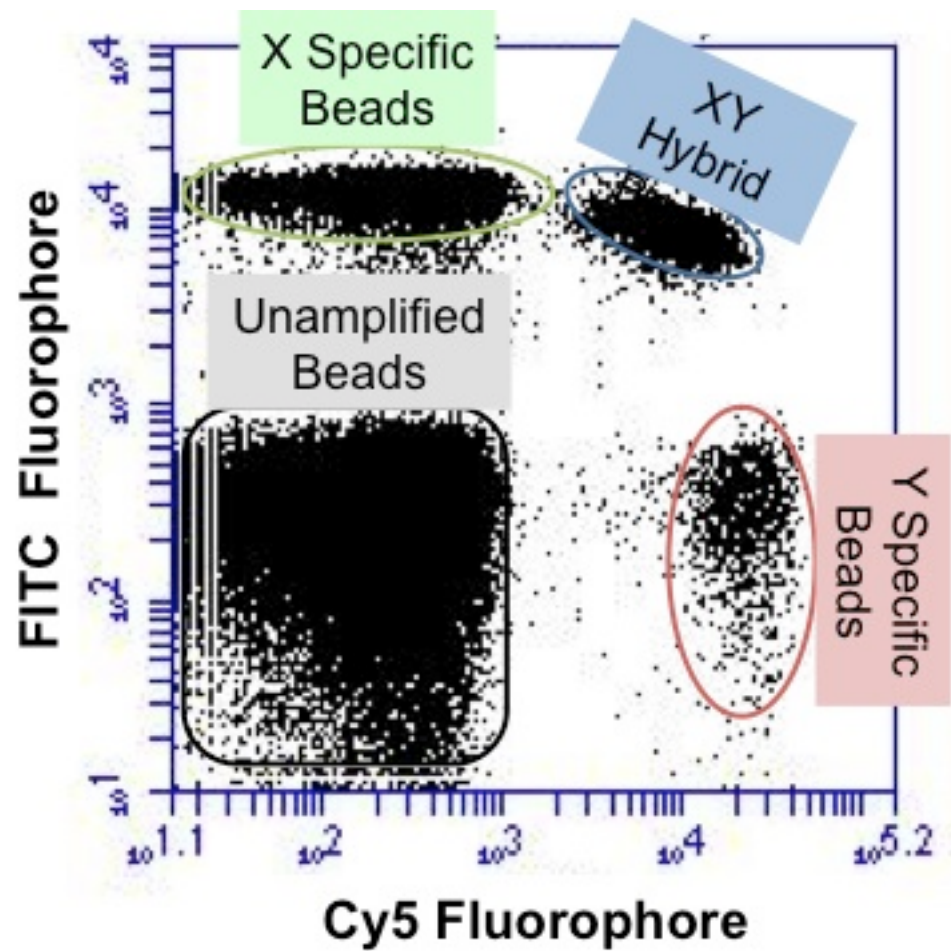

Supplement: S1 Fig — Beads are separated by a flow cytometer as negative (unamplified), AMELX positive, AMELY positive and double positives (AMELX and AMELY). The beads, positive only for a single signal type (AMELX, green beads, or AMELY, red beads) are used for calculations; beads with both AMELY and AMELY DNA (double positives, blue beads) are excluded from the analyses. (PDF) [file pone.0126501.s001.pdf]

Supporting Information File 2.

S2A Fig.

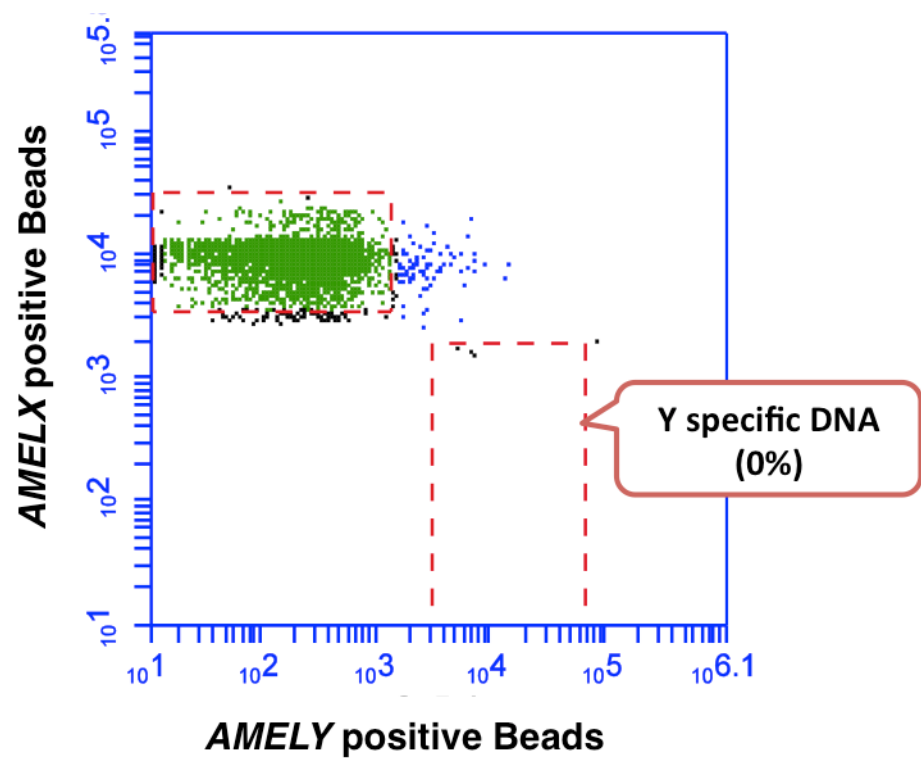

S2B Fig.

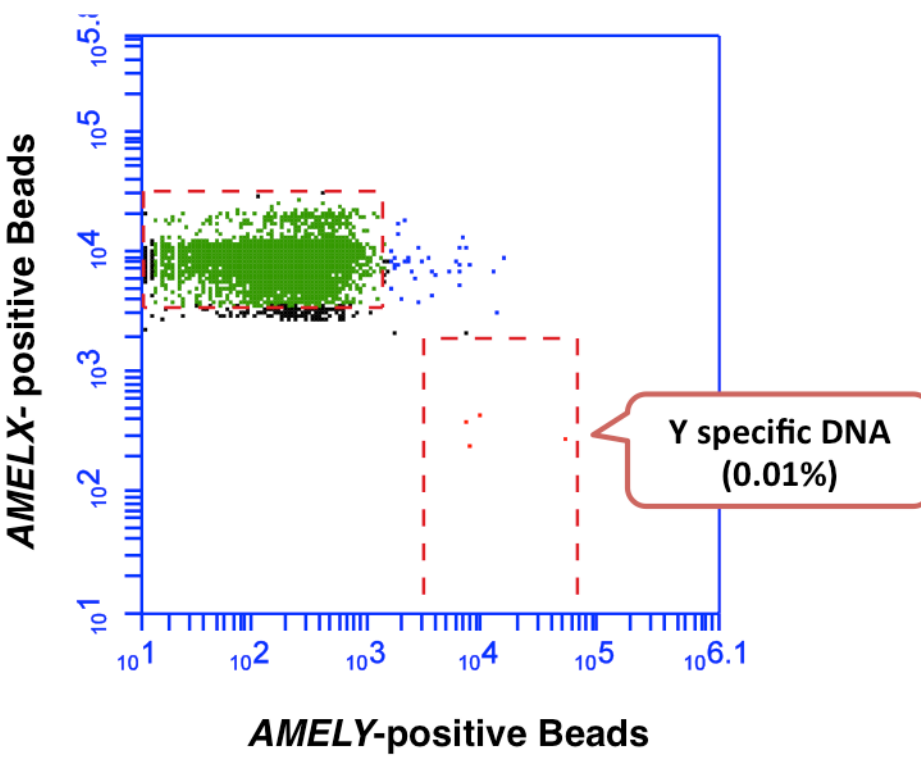

S2C Fig.

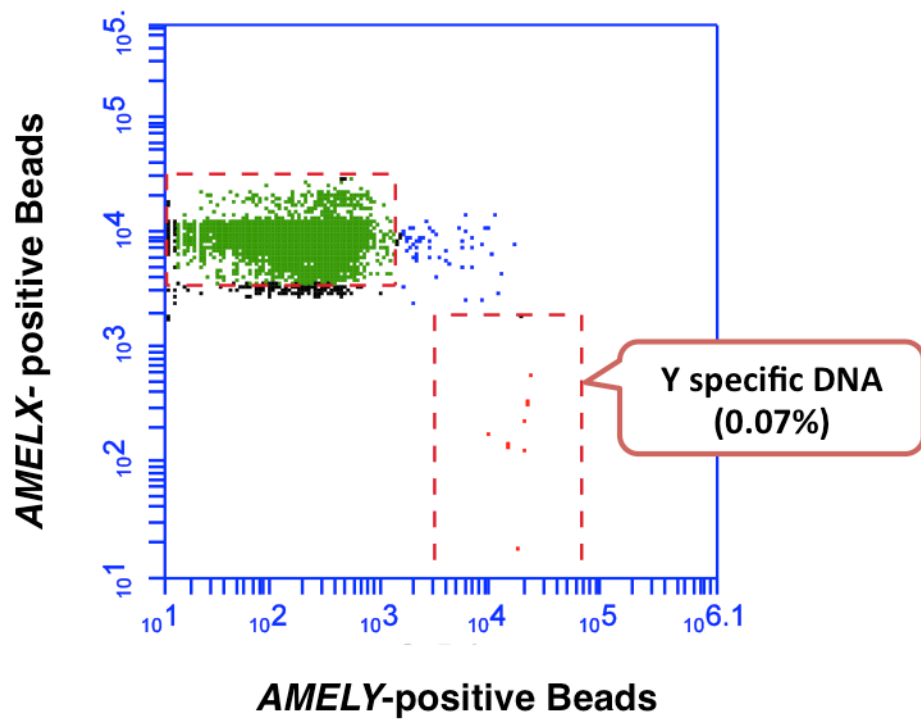

S2D Fig.

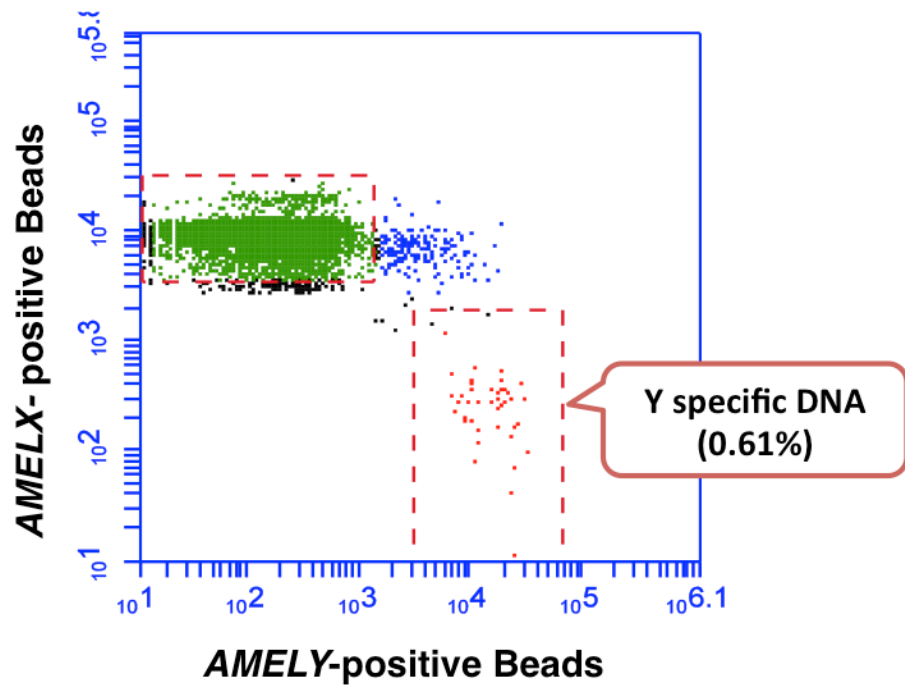

S2E Fig.

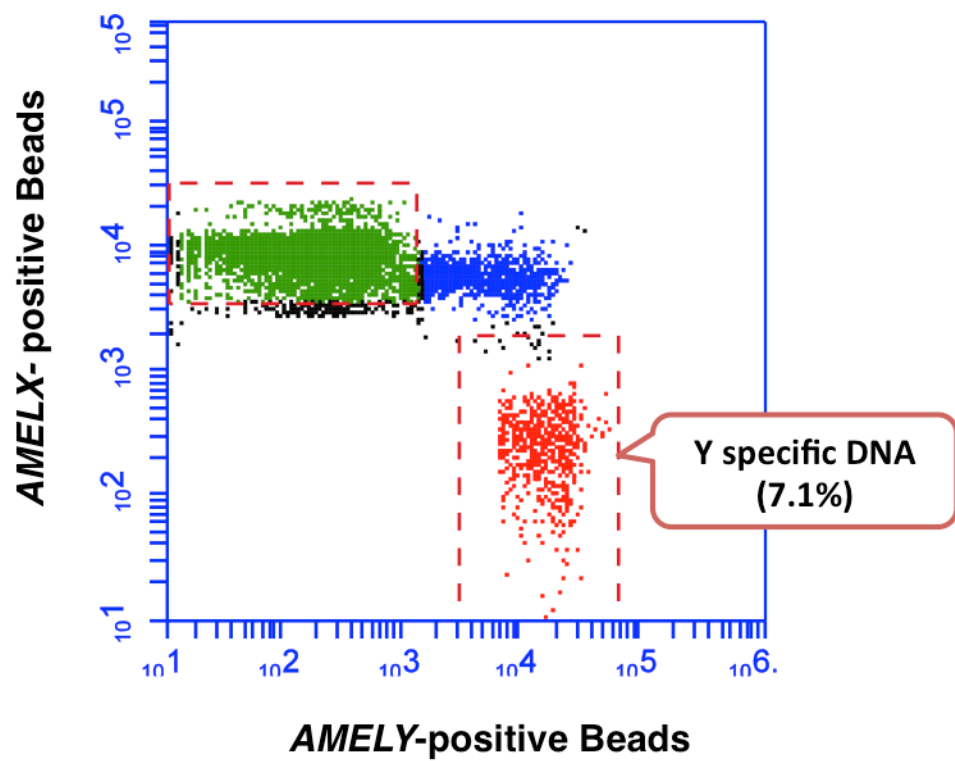

Supplement: S2 Fig — The relative AMELY positive fractions of serially diluted samples; (A) 0.0%, (B) 0.01%, (C) 0.1%, (D) 1%, and (E) 10%. (PDF) [file pone.0126501.s002.pdf]
